# Premonitory Urges and Their Link With Tic Severity in Children and Adolescents With Tic Disorders

**DOI:** 10.3389/fpsyt.2019.00569

**Published:** 2019-08-14

**Authors:** Maria Kyriazi, Efrosini Kalyva, Efthymia Vargiami, Konstantinos Krikonis, Dimitrios Zafeiriou

**Affiliations:** ^1^1st Department of Pediatrics, Hippokratio General Hospital, Aristotle University, Thessaloniki, Greece; ^2^Centre of Child and Adolescent Research and Development, Thessaloniki, Greece; ^3^DatAnalysis, Statistical Analysis and Design of Scientific Research, Ioannina, Greece

**Keywords:** tic disorders, premonitory urges, severity, tourette syndrome, Yale Global Tic Severity Scale

## Abstract

Tics wax and wane regarding their severity, while their expression is affected by non-motor sensory or cognitive elements that are mostly known as “premonitory urges.” Since premonitory urges are often used in non-pharmacological interventions to decrease tic severity, it is of interest in the present study to examine whether premonitory urges can actually predict tic severity. Fifty-two children and adolescents diagnosed with tics and Tourette syndrome (29 children with provisional tic disorder, 16 children with chronic motor tic disorder, and 7 children with Tourette syndrome) were included in the study. Their age ranged between 6 and 15.7 years (mean age 9 years and 2 months). All participants completed the YGTSS (Yale Global Tic Severity Scale) in order to assess tic severity and the Premonitory Urge for Tics Scale (PUTS) to measure premonitory urges (PU). Regression analysis revealed that PU were present at a higher rate in older subjects (>12 years of age) than in younger children and with a higher level of tic severity. Although the presence of PU was associated with tic severity across the entire age range, there was a stronger association between PU and tic severity in older children. A better insight into the pathophysiology of premonitory urges could possibly lead to the identification of new therapeutic modalities targeting the sensory initiators of tics in future research.

## Introduction

Tics are defined as sudden, rapid, recurrent, non-rhythmic motor movements or vocalizations usually appearing in bouts while waxing and waning in frequency, intensity, and manifestation. The most recent categorizations that emerged in the *Diagnostic and Statistical Manual of Mental Disorders, Fifth Edition* regarding motor disorders are developmental coordination disorder, stereotypic movement disorder, Tourette’s disorder, persistent (chronic) motor or vocal tic disorder, provisional tic disorder, other specified tic disorders, and unspecified tic disorders (1).

Tics are the most common movement disorders in childhood with transient tics affecting up to 10% of school-age children (2). Differences in the reported prevalence of chronic tic disorders from 0.5% to 4% (3–7) may be due to the fact that most cases of tics are mild and may be misdiagnosed or unrecognized by medical professionals (8). There is general consensus, however, that the male to female ratio is at least 2:1 or higher (9, 10), with prevalence rates of tic disorders in general ranging from 5.9% to 18% for males and from 2.9% to 11% for females (6).

Tic expression has been associated with non-motor elements (e.g., feelings of energy, tension, pressure, etc), either sensory or cognitive, that are somewhat difficult to categorize and have been named “premonitory urges” (11, 12).

The interacting systems involved in tic generation are primarily the SMA (supplementary motor area) which is implicated in pre-tic events (premonitory sensations) and secondarily the somatosensory and premotor cortex, which are implicated in tic execution in addition to classical neural pathways associated with voluntary movements (13).

The premonitory sensations that precede most tics are relieved by the tic itself (14). Some authors opine that premonitory sensations increase with age (15). The core feature of tics that distinguish them from other hyperkinesias, particularly myoclonus and chorea, is that they are suppressible for variable periods of time (from seconds to hours). The ability to suppress tics depends on the strength of premonitory sensations that have to be “released” sooner or later. Other authors claim that there is no relation between the perceived strength of premonitory sensations and patients’ ability to inhibit tics (16).

Only a few studies have looked at the relationship between premonitory urges and tic severity and reported contradictory findings. Woods, Piacentini, Himle, and Chang (17) found that increased awareness of premonitory sensations was linked to more severe tics, while Steinberg et al. (18) did not confirm this relationship. Moreover, although premonitory urges do occur in young children, they are often looked for in patients after the age of 10 (18). Therefore, the purpose of this study is to explore whether premonitory urges can predict tic severity regardless of age, since they are used in non-medical interventions as a tool to suppress tic expression.

## Methods

### Patients

The participants were children and adolescents diagnosed with tic disorder or Tourette syndrome in a tertiary neurodevelopmental center of a pediatric clinic who did not receive any medication and were not in remission of their tic symptoms. The diagnosis was made by a multidisciplinary team based on the criteria set by the Diagnostic and Statistical Manual of Mental Disorders, Fifth Edition (DSM-5) (provisional tic disorder, chronic motor or vocal tic disorder, and Tourette syndrome). The participants were 52 children and adolescents [29 (55.8%) children with provisional tic disorder, 16 (30.8%) children with chronic motor tic disorder, and 7 (13.5%) children with Tourette syndrome; none of the participants met the criteria for chronic vocal tic disorder]. Participants’ age ranged between 6 and 15.7 years (mean age 9 years and 2 months). Given the wide age range, the participants were assigned to one of the following two groups according to their age. Group A includes 39 children aged from 6 years to 11 years and 11 months, and Group B includes 13 children aged from 12 years to 15 years and 7 months.

### Tools

Clinicians—according to patients’ answers—completed the Yale Global Tic Severity Scale (YGTSS) (19) to assess tic severity and the Premonitory Urge for Tics Scale (PUTS) (17) to assess premonitory urges.

YGTSS is a semi-structured clinician-rated instrument of motor and phonic tic severity. The clinician initially notes the presence of motor and phonic tics based on child’s and parents’ reports over the previous week and behavioral observations. Following this, ratings are made for motor and phonic tics on five domains each: number, frequency, intensity, complexity, and interference. Each dimension is scored on a five-point scale, separately for vocal and motor tics. A total severity score is obtained by summing all scores across vocal and motor tics (range 0–50) or separately for vocal and motor tics. It also includes a separate impairment rating (score range 0–50). A global severity score is determined (0–100) by the sum of the total tics score and the impairment score (19, 20). The instrument has demonstrated good internal consistency and convergent and divergent validity (19, 20).

PUTS includes nine items designed to measure both sensory and mental phenomena. Each item is rated on a four-point ordinal scale (from 1 = not at all true to 4 = very much true). The total score is obtained by summing all 9 items (score range 9–36). Higher scores indicate the presence of more frequent pre-tic urges. PUTS has demonstrated high internal consistency and convergent validity (17).

Both scales were translated into the local language and back into English by experts proficient in both languages. A small pilot study was conducted with five children and their parents in order to ensure that all questions were comprehensive. No changes were needed, as can be seen from the Cronbach α scores. More specifically, Cronbach α for the subscales and the total score for YGTSS for the specific study ranged from 0.78 to 0.89 and was deemed satisfactory. The Cronbach α for the total PUTS score for the specific study was 0.83 and was deemed satisfactory.

### Procedure

The study has received ethics approval from the Bioethics Committee of the University. The participants were approached during their annual clinical visits, and they were asked if they would like to participate in the study. Out of the 65 patients who were approached initially 52 (80%) agreed to participate. Non-respondents offered mainly lack of time or the patients’ young age as an excuse not to participate. Parents signed informed consent forms, whereas patients offered their oral assent to participate. The assessment took place in the clinicians’ office, and the patients provided the responses.

The participants were older than 6 and able to read and understand the questions of both scales. The younger ones were given a reading comprehension test to ensure that they could understand the meaning of the question. In two cases that the patient was not able to fully comprehend a question the clinicians rephrased it and offered an example to help them provide the most appropriate answer.

### Statistical Analysis

Data were analyzed using SPSS.21 and distribution normality was tested using the appropriate measures. Stepwise linear regression was used to explore whether premonitory urges and age can be used to predict tic severity. Bivariate correlations were run for each age group separately to explore the relationship between premonitory urges and tic severity.

## Results

Analysis using χ^2^ revealed that there were no statistically significant differences between the two groups of tic disorders in terms of their age (χ^2^ = 1.48, df = 2, p = 0.478). (Please see **Table 1**).

**Table 1:** Percentages of children with TD by age and diagnosis.

Age	CMTD **N (%)**	Tourette **N (%)**	PTD **N (%)**
6-12	12 (30.8)	4 (10.2)	23 (59)
>12	4 (30.8)	3 (23.1)	6 (46.1)
Total	16 (30.8)	7 (13.5)	29 (55.7)

There were 38 male (73%) and 14 (27%) female participants. Analysis using χ^2^ revealed that there were no statistically significant gender differences between the two age groups [χ^2^ = 0.13, df = 1, p = 0.512]. (Please see **Table 2**).

**Table 2:** Percentages of children with TD by age and gender.

Age	Boys **N (%)**	Girls **N (%)**
6-12	28 (71.8)	11 (28.2)
>12	10 (76.9)	3 (23.1)
Total	38 (73.1)	14 (26.9)

Independent-samples t-test analysis revealed that there were statistically significant differences in the premonitory urges (PUTS) scores between the two age groups [t (50) = −1.84, p = 0.036]. Patients aged more than 12 years had significantly higher PUTS scores (M = 17.15, SD = 4.61) than children aged 6–12 (M = 14.18, SD = 5.19). Independent-samples t-test analysis revealed that there were statistically significant differences in the patients’ tic severity (YGTSS) scores between the two age groups [t (50) = −1.79, p = 0.040]. Patients aged more than 12 years had significantly higher YGTSS scores (M = 34.15, SD = 17.42) than children aged 6–12 (M = 24.82, SD = 15.85).

Stepwise multiple regression analysis was used to test if premonitory urges (PUTS) and age significantly predicted patients’ tic severity (YGTSS). The results of the regression indicated that premonitory urges explained 27% of the variance (R^2^ = 0.27, F(1,50) = 13.87, p = 0.000). It was found that premonitory urges significantly predicted tic severity (β = 0.466, p = 0.000), while age was excluded as a predictor of tic severity (β = 0.138, p = 0.292)

A bivariate correlation was run to determine the relationship between an individual’s PUTS and YGTSS scores for each of the two age groups. There was a moderate positive correlation between PUTS and YGTSS r(72) = 0.37, p = 0.022 for children aged 6–12 years and a strong positive correlation between PUTS and YGTSS r(72) = 0.65, p = 0.016 for children aged older than 12 years.

## Discussion

In the current study we could demonstrate that premonitory urges can predict almost 30% of the variance of tic severity, and this finding provides evidence for the importance of using premonitory urges as a tool to control and eventually eliminate tics using behavioral interventions. For example, habit reversal therapy is an active and effortful form of tic control where patients are trained to pay attention to premonitory urges to prevent expression of the corresponding tic, strengthening thus learned associations between interoceptive urge signals and inhibitory motor command. Patients who were aware early on of their premonitory urges were able to inhibit their tics more effectively (21), creating a model whereby people perceive their own voluntary actions as an internal signal in the presence of neural noise. Patients with effective tic inhibition may be better at reducing the level of internal noise, thus facilitating detection of their own intentions. It is important to note that perceptual discrimination between different classes of motor signals (e.g., those related to voluntary movements and those related to tics) may enhance even further the voluntary inhibitory control of tics, building on the developmentally inherent skill to discriminate between voluntary and involuntary movements (22). Therefore, the core deficit underlying tic disorders may extend beyond a simply overactive tic generator and represent a developmental delay in perceptual discrimination between true volition and premonitory urge.

It was shown that premonitory urges are an important predictor of tic severity, even when controlling for age, which means that although older children report more premonitory urges and present with more severe tics, younger children’s tic severity is also associated with their perceived premonitory urges. Therefore, age may have limited predictive value for tic severity (22), and it is therefore essential to stress the importance of training younger patients to recognize and verbally acknowledge their premonitory urges in an effort to suppress their tics more effectively. Although it is established that physiologically premonitory urges precede tic expression in all ages, they are often researched in relation to tic expression and suppression after the age of 10 (11, 12). This may be due either to methodological limitations (such as sample age and access) or to cognitive limitations that are believed to influence the young child’s ability to recognize and verbally express his/her own feelings.

In the study of Gulisano et al. (23) 95 patients (children and adolescents/young people) with Tourette syndrome were assessed. Their results showed that older children were more consistent in reporting premonitory urges than younger ones (i.e., PUTS scores increased with age), confirming also our findings. But they found no correlations between PUTS score and tic severity, in contrast to our findings.

The study of Kano et al. (24) also had similar findings with our study where the PUTS total scores were significantly correlated with the YGTSS subscales scores of number, complexity, and interference, which were identified also by Woods et al. (17).

Moreover Tinaz et al. (25) found a positive correlation with both the urge to tic and tic severity in patients in Tourette syndrome.

It should be stressed that patients who learn to focus on their premonitory urges in order to control their tics are likely to experience an initial increase in their tics. The role of attention in generation and perception of involuntary movements is increasingly recognized. Patients with Tourette syndrome who pay attention to their tics present with more tics (16). If children and/or adolescents and their parents learn to deal with this sudden, but temporary, increase of the symptoms they are trying to suppress, it could lead to better therapeutic results. Clinicians can also use techniques that are effective with children with obsessive-compulsive symptoms or attention-deficit/hyperactivity disorder to deal with tic symptomatology since the comorbidity is high and there seems to be an underlying effect of the presence of premonitory urges (26) that needs to be further explored.

Some drawbacks of our study need to be addressed. There was no age of onset for PU reported because as Cavanna et al. (27) discussed, it is likely that patients become aware of their urges to tic through a maturational process, which is largely independent of tic onset (16, 17) and could be the expression of a transition from simple sensory perceptions to fully fledged consciousness (27). Greene et al. (28) reported that determining the date of tic onset is difficult as there is no clinical gold standard for doing so. There are some tics like frequent blinking that the parent could have overlooked may be a tic. However, there is still the possibility that other past tics may have gone unnoticed, which is an inevitable limitation of studying this population.

Also, from the very start of the study, we have restricted the study sample to treatment-naive patients without any comorbidities, thus limiting the range of various scores.

Despite these limitations, the present study provides significant insights into premonitory urges and tic severity and is the first study involving children in our country. The current results, that premonitory urges can predict tic severity—although the level of PU was associated with tic severity across the entire age range, there was a stronger association between PU and tic severity for the older children—suggest that further research on premonitory urges in relation to tics is warranted for better understanding the role that premonitory urges play in the maintenance of tic disorder.

## Conclusions

This study solidified the speculation that premonitory urges can account for a significant variance of tic severity irrespective of the child’s age. Therefore, it is important to use active tic control techniques that teach children ways to detect their premonitory urges in order to eventually suppress their tics. This suggestion is in line with European guidelines that propose behavioral interventions as the most effective way to treat tics followed by medication only in case that the former is not working. It is also likely that a developmental delay in perceptual discrimination between true volition and premonitory urge influences the child’s ability to recognize premonitory urges, and this limitation could be partially overcome through physiological measures to detect premonitory urges.

## Data Availability

The datasets generated for this study are available on request to the corresponding author.

## Ethics Statement

The study was approved by the Bioethics Committee of Aristotle University of Thessaloniki, parents signed informed consent forms whereas patients offered their oral assent to participate. This study was performed in accordance with the Declaration of Helsinki.

## Author Contributions

All authors participated in a meaningful way in the preparation of the manuscript (in the design or conceptualization of study, interpretation of data, or drafting the manuscript). All authors read and approved the final manuscript.

## Conflict of Interest Statement

The authors declare that the research was conducted in the absence of any commercial or financial relationships that could be construed as a potential conflict of interest.

## Abbreviations

YGTSS, Yale Global Tic Severity Scale; PUTS, Premonitory Urge for Tics Scale; SMA, supplementary motor area.

## References

[1] American Psychiatric Association DSM 5. (2013). 10.1176/appi.books.9780890425596.744053

[2] Caurín B Serrano M Fernández-Alvarez E Campistol J Pérez-Dueñas B Environmental circumstances influencing tic expression in children. Eur J Paediatr Neurol (2014) 18:157–62. 10.1016/j.ejpn.2013.10.002 24210363

[3] McNaught KS Mink JW Advances in understanding and treatment of Tourette syndrome. Nat Rev Neurol (2011) 7:667–76. 10.1038/nrneurol.2011.167 22064610

[4] Suarez A Tics en Pediatrıa. Pediatr Integral (2008) 10:989–94.

[5] Scahill L Sukhodolsky D Williams S Leckman JF The public health importance of tics and tic disorders. Adv Neurol (2005) 96:240–8 16383223

[6] Khalifa N von Knorring AL Prevalence of tic disorders and Tourette syndrome in a Swedish school population. Dev Med Child Neurol (2003) 45:315–9. 10.1111/j.1469-8749.2003.tb00402.x 12729145

[7] Olson S Neurobiology. Making sense of Tourette’s. Science (2004) 305:1390–2. 10.1126/science.305.5689.1390 15353772

[8] Neuner I Ludolph A Tics and Tourette’s syndrome throughout the life span. Nervenarzt (2009) 80:1377–87. 10.1007/s00115-009-2807-0 19855949

[9] Prado HS Rosário MC Lee J Hounie AG Shavitt RG Miguel EC Sensory phenomena in obsessive-compulsive disorder and tic disorders: a review of the literature. CNS Spectr (2008) 13:425–32. 10.1017/S1092852900016606 18496480

[10] Leckman JF Walker D Cohen D Premonitory urges in Tourette’s syndrome. Am J Psychiatry (1993) 150:98–101. 10.1176/ajp.150.1.98 8417589

[11] Specht MW Woods DW Nicotra CM Kelly LM Ricketts EJ Conelea CA Effects of tic suppression: ability to suppress, rebound, negative reinforcement, and habituation to the premonitory urge. Behav Res Ther (2012) 51:24–30. 10.1016/j.brat.2012.09.009 23168328

[12] Robertson MM The prevalence and epidemiology of Gilles de la Tourette syndrome. Part 1: the epidemiological and prevalence studies. J Psychosom Res (2008) 65:461–72. 10.1016/j.jpsychores.2008.03.006 18940377

[13] Woods DW Himle MB Miltenberger RG Carr JE Osmon DC Karsten AM Durability, negative impact, and neuropsychological predictors of tic suppression in children with chronic tic disorders. J Abnorm Child Psychol (2008) 36:237–45. 10.1007/s10802-007-9173-9 17717739

[14] Wang Z Maia TV Marsh R Colibazzi T Gerber A Peterson BS The neural circuits that generate tics in Tourette’s syndrome. Am J Psychiat (2011) 168:1326–37. 10.1176/appi.ajp.2011.09111692 PMC4246702 21955933

[15] Kwak C Dat Vuong K Jankovic J Premonitory sensory phenomenon in Tourette’s syndrome. Mov Disord (2003) 18:1530–3. 10.1002/mds.10618 14673893

[16] Banaschewski T Woerner W Rothenberger A Premonitory sensory phenomena and suppressibility of tics in Tourette syndrome: developmental aspects in children and adolescents. Dev Med Child Neurol (2003) 45:700–3. 10.1111/j.1469-8749.2003.tb00873.x 14515942

[17] Woods DW Piacentini J Himle MB Chang S Premonitory Urge for Tics Scale (PUTS): initial psychometric results and examination of the premonitory urge phenomenon in youths with tic disorders. J Dev Behav Pediatr (2005) 26:397–403. 10.1097/00004703-200512000-00001 16344654

[18] Steinberg T Shmuel Baruch S Harush A Dar R Woods D Piacentini J Tic disorders and the premonitory urge. J Neural Transm (2010) 117:277–84. 10.1007/s00702-009-0353-3 PMC2809309 20033236

[19] Leckman JF Riddle MA Hardin MT Ort SI Swartz KL Stevenson J The Yale Global tic severity scale: initial testing of a clinician-rated scale of tic severity. J Am Acad Child Adolesc Psychiatry (1989) 28:566–73. 10.1097/00004583-198907000-00015 2768151

[20] Storch EA Murphy TK Fernandez M Krishnan M Geffken GR Kellgren AR Factor-analytic study of the Yale Global Tic Severity Scale. Psychiatry Res (2007) 149:231–7. 10.1016/j.psychres.2006.03.017 17150256

[21] Ganos C Garrido A Navalpotro-Gómez I Ricciardi L Martino D Edwards MJ Premonitory urge to tic in Tourette’s is associated with interoceptive awareness. Mov Disord (2015) 30:1198–202. 10.1002/mds.26228 25879819

[22] Ganos C Rothwell J Haggard P Voluntary inhibitory motor control over involuntary tic movements. Mov Disord (2018) 33 (6), 937–946. 10.1002/mds.27479 29508917

[23] Gulisano M Calì P Palermo F Robertson M Rizzo R Premonitory urges in patients with Gilles de la Tourette syndrome: an Italian translation and a 7-year follow-up. J Child Adolesc Psychopharmacol (2015) 25:810–6. 10.1089/cap.2014.0154 26288345

[24] Kano Y Matsuda N Nonaka M Fujio M Kuwabara H Kono T Sensory phenomena related to tics, obsessive-compulsive symptoms, and global functioning in Tourette syndrome. Compr Psychiatry (2015) 62:141–6. 10.1016/j.comppsych.2015.07.006 26343478

[25] Tinaz S Malone P Hallett M Horovitz SG, Role of the right dorsal anterior insula in the urge to tic in Tourette syndrome. Mov Disord (2015) 30:1190–7. 10.1002/mds.26230 PMC5088605 25855089

[26] Leckman JF Bloch MH King RA Scahill L Phenomenology of tics and natural history of tic disorders. Adv Neurol (2006) 99:1–16. 16536348

[27] Cavanna AE Black KJ Hallett M Voon V Neurobiology of the premonitory urge in Tourette’s syndrome: pathophysiology and treatment implications. J Neuropsychiatry Clin Neurosci (2017) 29(2):95104. 10.1176/appi.neuropsych.16070141 PMC5409107 28121259

[28] Greene D Koller JM Robichaux-Viehoever A Bihun EC Schlaggar BL Black KJ Reward enhances tic suppression in children within months of tic disorder onset. Dev Cogn Neurosci (2015) 11, 65–74. 10.1016/j.dcn.2014.08.005 25220075 PMC4323948

